# A Rare Case of Gastrointestinal Amyloidosis Due to Monoclonal Gammopathy of Undetermined Significance

**DOI:** 10.7759/cureus.37953

**Published:** 2023-04-21

**Authors:** Sarah Singh, Guru Gopireddy, Scott Naum, Michael P Iannetti

**Affiliations:** 1 Internal Medicine, Camden Clark Medical Center, Parkersburg, USA; 2 Gastroenterology, Camden Clark Medical Center, Parkersburg, USA

**Keywords:** stomach, duodenum, amyloidosis, monoclonal gammopathy of undetermined significance (mgus), hematology, gastroenterology

## Abstract

Amyloidosis of the gastrointestinal (GI) tract is caused by the deposition of fibrils made of serum proteins into extracellular spaces. It is an uncommon disease with a poor prognosis, requiring prompt diagnosis and treatment. Treatment for amyloid light chain (AL)-type amyloidosis involves supportive care as well as addressing any underlying plasma cell dyscrasias. We present the case of a 64-year-old female diagnosed with AL-type GI amyloidosis with associated monoclonal gammopathy of undetermined significance. Unfortunately, the treatment was initiated nine months after the initial presentation, and she died one month later. Awareness of GI amyloidosis may allow for faster diagnosis and treatment in future patients.

## Introduction

Amyloidosis of the gastrointestinal (GI) tract may be caused by localized or systemic amyloidosis. Primary systemic amyloidosis is associated with monoclonal light chains (AL amyloidosis), secondary systemic amyloidosis is associated with serum amyloid (AA amyloidosis), and dialysis-related systemic amyloidosis is associated with beta-2-microglobulin (Aβ2M amyloidosis) [1]. AL amyloidosis is usually an acquired, systemic amyloidosis commonly caused by hematologic malignancies and is known to have a poorer prognosis. It is also most commonly associated with GI manifestations [1-4]. In Western countries, AL amyloidosis has an incidence of one case per 100,000 person-years [5]. In this disease, amyloid proteins invade mucosal or neuromuscular tissues, leading to fragile blood vessels, decreased gut wall compliance, and symptoms resembling fibrotic liver disease [1,3]. Symptomatic GI amyloidosis usually presents with one of four syndromes, namely, GI bleeding, malabsorption, protein-losing gastroenteropathy, or GI dysmotility [1,3]. The most common sites involve the duodenum, stomach, colorectum, and esophagus [6]. Nonspecific symptoms of amyloidosis also include anorexia, fatigue, weight loss, and lightheadedness [1]. Diagnosis of GI amyloidosis requires a tissue biopsy with positive staining of amyloid by Congo red [1,3]. Biopsies taken from the duodenum have been noted to have the highest yield [1]. Once diagnosed, urine and serum electrophoresis should be conducted to evaluate for plasma cell dyscrasias. Treatment typically involves supportive care, including volume resuscitation, dietary modifications, pro-kinetic or antiemetic medications, surgery, and treatment of underlying malignancy [1-3]. For AL amyloidosis, as it is commonly caused by plasma cell dyscrasias, autologous stem cell transplant or chemotherapy with a combination of melphalan, bortezomib, and dexamethasone is associated with increased survival [1,2,5]. The prognosis of patients with amyloidosis and GI involvement appears worse than those without GI involvement [1].

This case was previously presented as an abstract at the American College of Physicians West Virginia Chapter Meeting on November 5, 2021, as well as at the American College of Gastroenterology National Conference on October 25, 2022.

## Case presentation

**Figure 1:**
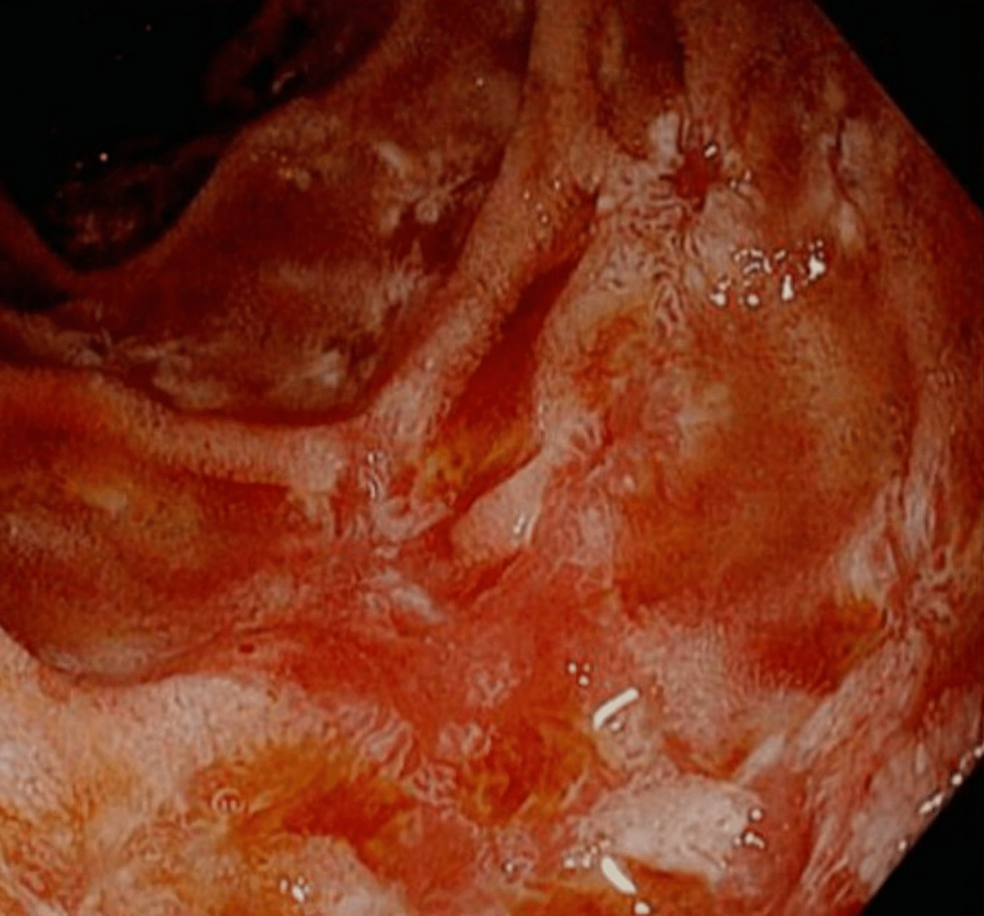
Lining of the first portion of the duodenum.

**Figure 2:**
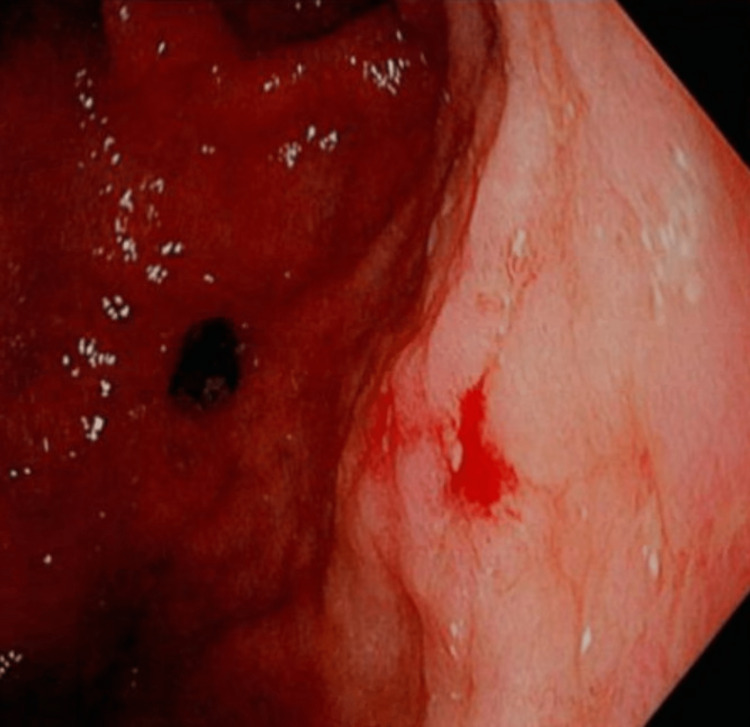
Lining of the stomach.

A 64-year-old female with a past medical history significant for hypertension, chronic obstructive pulmonary disease, systolic congestive heart failure, depression, gastroesophageal reflux disease, hyperlipidemia, hypothyroidism, and anxiety presented to the emergency department (ED) complaining of dyspnea, dysuria, fatigue, and right lower quadrant abdominal pain. Workup in the ED was significant for an alkaline phosphatase of 188 U/L (45-115 U/L), aspartate aminotransferase of 67 U/L (8-45 U/L), white blood cell count of 21.4 × 10^3^/µL (3.7-11.0 × 10^3^/µL), platelet count 639 × 10^3^/µL (150-400 × 10^3^/µL), and hemoglobin 8.6 g/dL (13.4-17.5 g/dL). Abdominal CT demonstrated retroperitoneal adenopathy and omental findings concerning for metastatic disease or carcinomatosis. An esophagogastroduodenoscopy (EGD) revealed multiple gastric and duodenal ulcers (Figures 1, 2).

Pathology reports from the duodenum and antrum of the stomach revealed chronic inflammation, giant cell reaction, and ischemic-type necrosis.

**Figure 3:**
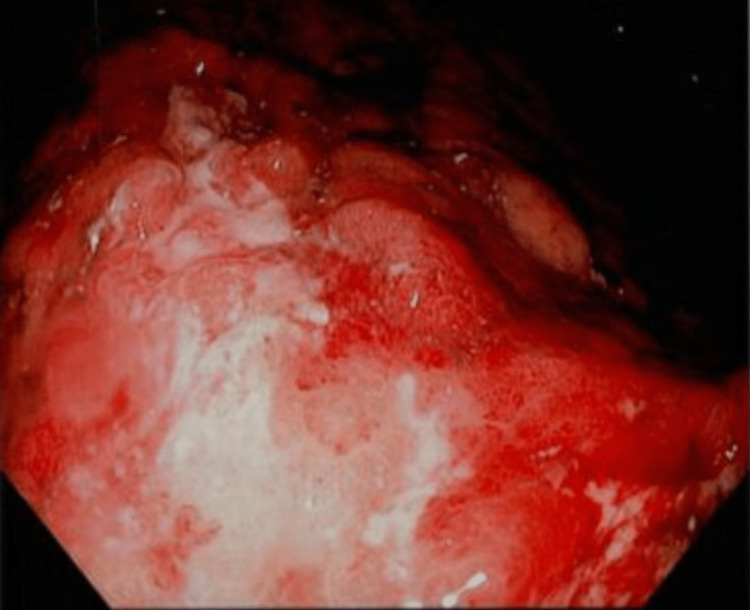
Lining of the second portion of the duodenum.

**Figure 4:**
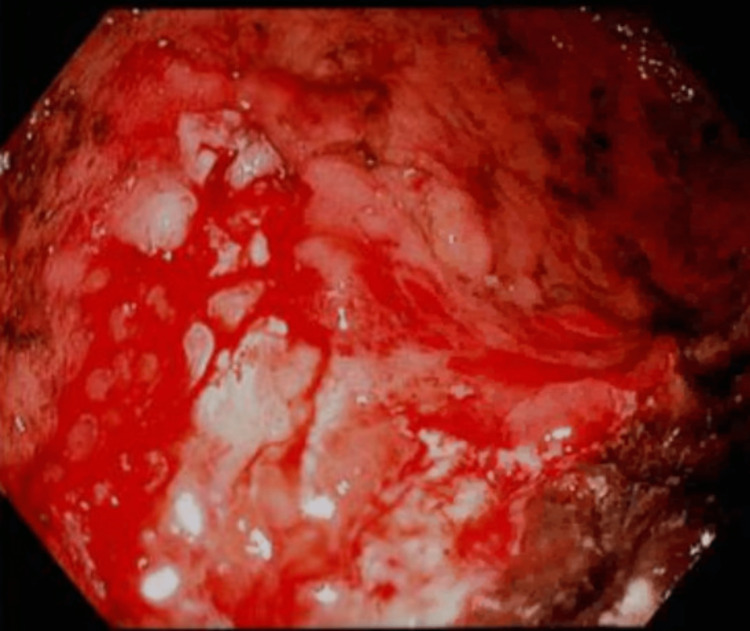
Lining of the second portion of the duodenum.

She was discharged and presented to the ED five months later complaining of hematemesis, weight loss, melanotic stool, and right upper quadrant abdominal pain. Repeat abdominal CT showed diffuse carcinomatosis with omental caking, signs of hemorrhage, and increased adenopathy. Repeat EGD revealed malignant-appearing, non-bleeding ulcerations in the body of the stomach infiltrating into the proximal stomach with active duodenitis in the first and second portions of the duodenum (Figures 3, 4).

**Figure 5:**
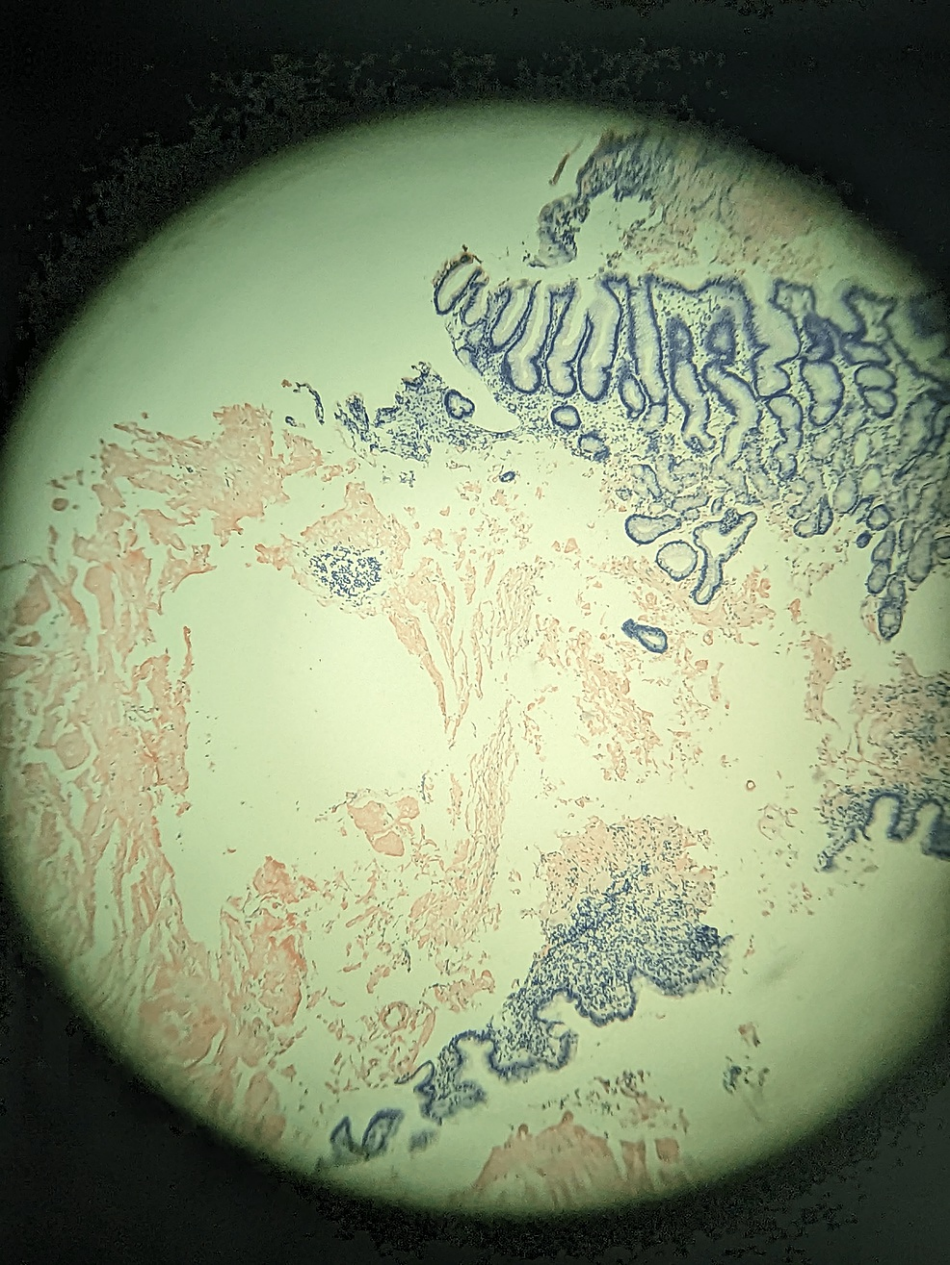
Gastric antrum with Congo red staining.

**Figure 6:**
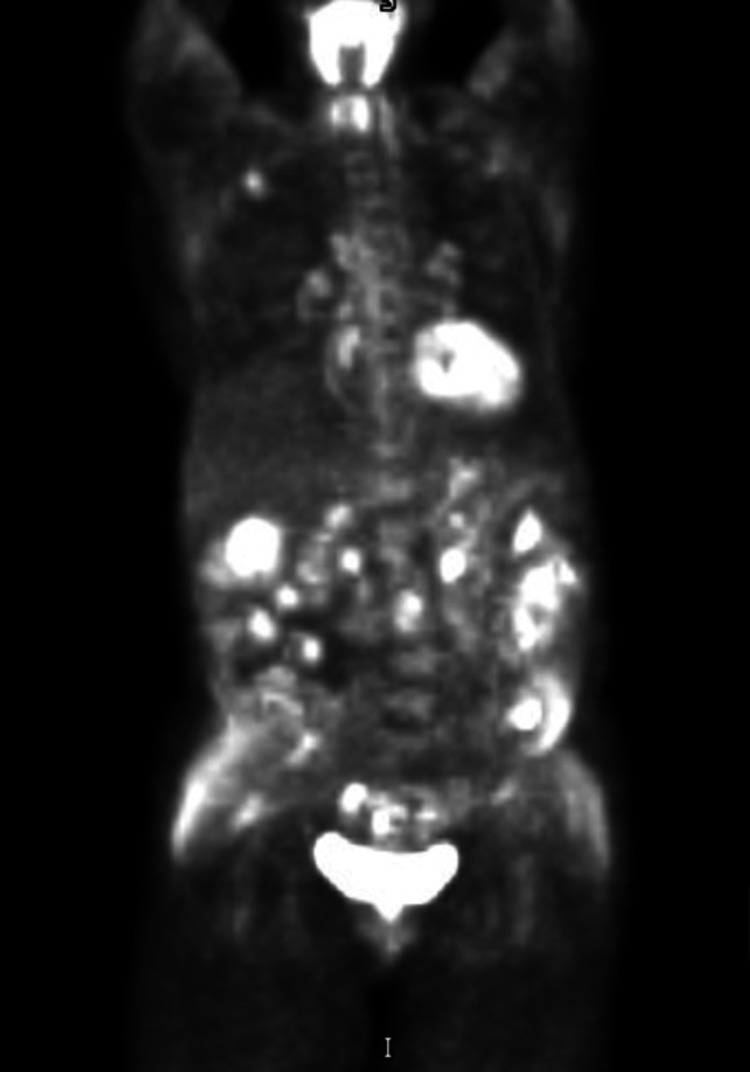
Positron emission tomography/computed tomography scan.

Repeat biopsies obtained from the lower body of the stomach and the second part of the duodenum revealed amorphous proteinaceous distribution suggestive of amyloidosis. Further analysis with Congo red staining was positive for amyloidosis with light chain deposition, indicating AL kappa-type amyloidosis (Figure 5). Serum protein electrophoresis testing was negative; however, urine protein electrophoresis demonstrated an M spike which was confirmed with immunotyping. Urine was positive for kappa light chains (Bence-Jones protein). Further workup with an echocardiogram demonstrated a left ventricular ejection fraction of 65% but a normal septum. A bone marrow biopsy was also conducted and showed normocellular hematopoietic marrow with a low-level population of monoclonal kappa-restricted plasma cells, consistent with monoclonal gammopathy of undetermined significance (MGUS). Positron emission tomography/computed tomography demonstrated moderate irregular gastric wall thickening that was faintly metabolically active, concerning for intraperitoneal spread (Figure 6). Our patient was then diagnosed with light chain deposition disease/amyloid kappa chain with multiorgan involvement.

She was due to start VRd (bortezomib, lenalidomide, and dexamethasone) induction therapy and was considered for an autologous bone marrow transplant. However, before beginning therapy, she presented to the ED again complaining of dyspnea and tongue swelling. Chest and abdominal CT demonstrated an increase in carcinomatosis and adenopathy involving the retroperitoneum and mesenteric regions. She was started on CyBorD (cyclophosphamide, bortezomib, and dexamethasone) nine months after her initial ED visit. She died at home one month later.

## Discussion

Our patient initially presented with dyspnea, dysuria, fatigue, and right lower quadrant pain but later also presented with hematemesis, weight loss, melanotic stool, and tongue swelling. These later presentations signified manifestations of her disease. From biopsies taken from bone marrow and GI tract, she was diagnosed with AL amyloidosis of the GI tract with multiorgan involvement and associated MGUS.

MGUS is an asymptomatic plasma cell disorder [7], and, as stated by Dhodapkar [8], it is genetically more similar to a multiple myeloma cell than a normal plasma cell. This raises concern for progression to multiple myeloma. Those with MGUS are also at risk of developing light-chain amyloidosis [9]. AL amyloidosis typically presents with symptoms relating to the heart, kidneys, and nervous system [9].

Case reports concerning GI amyloidosis have been reported on multiple myeloma, MGUS, but have not been associated with plasma cell dyscrasia [10-13]. Two other case reports described findings consistent with MGUS [14,15]. These patients presented at different stages of their disease, ranging from having an incidental finding [11,15] to presenting with severe complications such as hematemesis, anemia, and weight loss [10,12-14]. Furthermore, four of these six patients improved with treatment [10,11,14,15] while another two succumbed to the disease [12,13]. Future studies should focus on recognizing early signs and symptoms of AL amyloidosis and its many manifestations to avoid this outcome in future patients.

## Conclusions

This case illustrates that, while rare, clinicians must be aware of signs and symptoms suspicious for amyloidosis of the GI tract. Due to the progressive nature of the disease, early diagnosis is imperative for effective treatment and improved patient outcomes. Early follow-up of abnormal testing, which was missed in this case, can augment early diagnosis.
